# Novel Molecular Markers of Malignancy in Histologically Normal and Benign Breast

**DOI:** 10.4061/2011/489064

**Published:** 2011-07-10

**Authors:** Aejaz Nasir, Dung-Tsa Chen, Mike Gruidl, Evita B. Henderson-Jackson, Chinnambally Venkataramu, Susan M. McCarthy, Heyoung L. McBride, Eleanor Harris, Nazanin Khakpour, Timothy J. Yeatman

**Affiliations:** ^1^Department of Anatomic Pathology, Moffitt Cancer Center & Research Institute, Tampa, FL 33612, USA; ^2^Department of M2Gen Pathology, Moffitt Cancer Center & Research Institute, Tampa, FL 33612, USA; ^3^Department of Experimental Therapeutics, Moffitt Cancer Center & Research Institute, Tampa, FL 33612, USA; ^4^Oncology Biomarker Sciences Group, Diagnostic & Experimental Medicine, Lilly Research Laboratories, Eli Lilly & Company, Indianapolis, IN 46285, USA; ^5^Department of Biostatistics, Moffitt Cancer Center & Research Institute, Tampa, FL 33612, USA; ^6^Department of Molecular Oncology, Moffitt Cancer Center & Research Institute, Tampa, FL 33612, USA; ^7^Department of Pathology & Laboratory Medicine, School of Medicine, University of South Florida, Tampa, FL 33620, USA; ^8^Department of Surgery, Moffitt Cancer Center & Research Institute, Tampa, FL 33612, USA; ^9^Department of Radiation Oncology, Moffitt Cancer Center & Research Institute, Tampa, FL 33612, USA

## Abstract

To detect the molecular changes of malignancy in histologically normal breast (HNB) tissues, we recently developed a novel 117-gene-malignancy-signature. Here we report validation of our leading malignancy-risk-genes, topoisomerase-2-alpha (TOP2A), minichromosome-maintenance-protein-2 (MCM2) and “budding-uninhibited-by-benzimidazoles-1-homolog-beta” (BUB1B) at the protein level. Using our 117-gene malignancy-signature, we classified 18 fresh-frozen HNB tissues from 18 adult female breast cancer patients into HNB-tissues with low-grade (HNB-LGMA; N = 9) and high-grade molecular abnormality (HNB-HGMA; N = 9). Archival sections of additional HNB tissues from these patients, and invasive ductal carcinoma (IDC) tissues from six other patients were immunostained for these biomarkers. TOP2A/MCM2 expression was assessed as staining index (%) and BUB1B expression as *H*-scores (0–300). Increasing TOP2A, MCM2, and BUB1B protein expression from HNB-LGMA to HNB-HGMA tissues to IDCs validated our microarray-based molecular classification of HNB tissues by immunohistochemistry. We also demonstrated an increasing expression of TOP2A protein on an independent test set of HNB/benign/reductionmammoplasties, atypical-ductal-hyperplasia with and without synchronous breast cancer, DCIS and IDC tissues using a custom tissue microarray (TMA). In conclusion, TOP2A, MCM2, and BUB1B proteins are potential molecular biomarkers of malignancy in histologically normal and benign breast tissues. Larger-scale clinical validation studies are needed to further evaluate the clinical utility of these molecular biomarkers.

## 1. Introduction

Despite recent advances in biomarker discovery, no clinically proven biomarkers of increased breast cancer risk have been identified and validated in histologically normal breast. However, there is increasing evidence in the current literature for the presence of specific genetic abnormalities in histologically normal breast tissue in patients with and without breast cancer [1–10]. Such genetic abnormalities are often common to the tumor and their matched histologically normal breast tissues, suggesting their association with subsequent development of breast cancer in those patients. Whether such molecular abnormalities are the cause or the effect of the development of breast cancer is largely unknown. Also the degree of expression and microanatomical distribution of these molecular abnormalities in histologically normal/benign breast tissues is still poorly defined. 

In order to elucidate the molecular changes of malignancy in HNB tissues, we used the Affymetrix platform to profile a large prospective series of fresh-frozen HNB tissues and invasive ductal breast carcinomas (IDCs). Based on the differential expression of a number of IDC-specific genes in HNB tissues, we developed a novel 117-gene malignancy signature for molecular classification of HNB tissues into two subsets that we designated as those with high-grade and low-grade molecular abnormalities. We subsequently validated our microarray data on HNB tissues using real-time PCR (qPCR) [11] and demonstrated additional utility of our malignancy signature by cross-validation analyses on publically available breast data sets. 

Many of the genes in our 117-gene signature were “proliferation genes.” Some of these “proliferation genes” (TOP2A, MCM2, and BUB1B) are also important targets for breast cancer therapy. Here we report cross-platform validation of these 3 genes as our leading candidate malignancy genes at the protein level. We used immunohistochemistry on a new set of archival sections of HNB tissues from 18 breast cancer/DCIS/prophylactic mastectomy patients whose breast tissues (cancer and normal) were used to derive our 117-gene malignancy signature in the original microarray experiment. Since the cells lining the terminal duct lobular units (TDLUs) are thought to be the precursor cells of breast cancer [12], in this cross-platform (microarray to IHC) validation analysis we focused primarily on the immunohistochemical expression of TOP2A, MCM2, and BUB1B in the histologically normal TDLUs, although we believe that these proteins may also be useful in the molecular stratification of benign breast disease and premalignant breast lesions such as atypical ductal hyperplasia (ADH). 

## 2. Materials and Methods

### 2.1. Patients and Specimens

**Figure 1:**
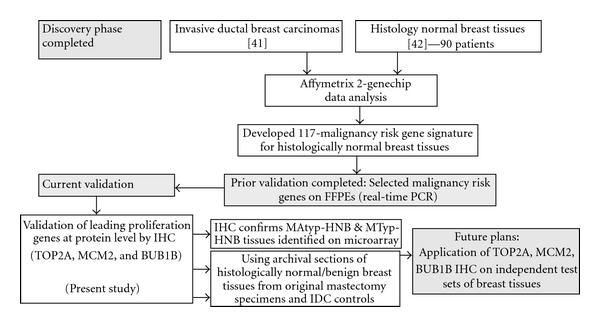
Molecular markers of malignancy in histologically normal breast tissues. Context and evolution of our prospective experimental plan.

This study was carried out under approval by the Institutional Review Board (IRB) at the University of South Florida, Tampa, FL. It included 24 adult female patients who underwent mastectomy for their breast cancers at Moffitt Cancer Center (MCC), Tampa, FL between 2002 and 2005. Eighteen of these patients had fresh-frozen histologically normal breast (HNB) tissues previously analyzed using Affymetrix Plus 2.0 Gene chip to develop a 117 gene signature to be used for molecular classification of histologically normal breast tissues. Based on the expression levels of 117-genes in our malignancy signature (Figure 1), these 18 specimens were classified as HNB tissues with high-grade and low-grade molecular abnormalities (HNB-HGMA; *N*=9 and HNB-LGMA; *N*=9). Mean ages for patients with HNB-HGMA and HNB-LGMA were 50 and 55 years, respectively. Pertinent clinicopathologic data, based on information available from MCC and Contributing Institutions' Surgical Pathology reports, electronic patient records, MCC Cancer Registry, and retrospective review of all available H&E slides from MCC Pathology Archives and outside institutions, is summarized in Table 1. 

All available formalin fixed, paraffin-embedded (FFPE) sections from the mastectomies of the study patients (*N* = 18) were reviewed by an experienced breast pathologist (AN) to select HNB tissue blocks for immunohistochemical validation of 3 of our leading malignancy-risk genes (TOP2A, BUB1B, and MCM2). The selection of FFPE block representative of each HNB tissue was based on the presence of maximum number of histologically normal terminal duct lobule units (TDLUs) on a single H&E. stained section among all of the archival sections reviewed from that patient. Archival tumor sections from 6 other adult female patients (mean patient age: 69 years) with IDCs (Cases  1–6; Table 1) were selected as positive tissue controls to validate the immunohistochemical expression of TOP2A, MCM2, and BUB1B protein on archival sections of HNB tissues.  Table 2 compares ages for the 3 patient groups in this analysis. 

### 2.2. TOP2A, MCM2, and BUB1B Protein Immunohistochemistry

Five-micron thick serial FFPE sections from each selected IDC (*N* = 6), HNB-HGMA (*N* = 9), and HNB-LGMA (*N* = 9) tissue block were stained with H&E, and for TOP2A, MCM2, and BUB1B protein proteins, using immunohistochemical (IHC) protocols optimized in the Tissue Core Laboratory at our institute (AN). The IHC staining was carried out using a Ventana Discovery XT automated system (Ventana Medical Systems, Tucson, AZ, USA) as per manufacturer's protocol with proprietary reagents. Briefly, slides were deparaffinized on the automated system with EZ Prep solution (Ventana). Enzymatic retrieval was used with Protease 1 solution (Ventana). 

The mouse monoclonal antibody that reacts with human TOP2A protein (#MS-1819-SO, Neomarkers) was used at a 1 : 50 concentration in Dako antibody diluent and incubated for 60 min. The mouse monoclonal antibody that reacts with human MCM2 protein (#MS1726PO, Neomarkers) was used at a 1 : 100 concentration in Dako antibody diluent and incubated for 4 hours. The BUB1B staining required a 4-minute treatment with Ventana Protease 1 prior to a 60-minute incubation with the BUB1B antibody (diluted 1 : 100, Abcam, #AB54894). The Ventana Omni Anti-Mouse HRP Secondary Antibody (prediluted) was used for 16 min. The detection system used was the Ventana Omni UltraMap kit, and slides were then counterstained with hematoxylin. Slides were dehydrated and cover-slipped as per standard tissue core laboratory protocol. 

### 2.3. Control Tissues Used for Immunohistochemical Optimization and Test Runs

Positive control tissues that were used for optimization of the above IHC protocols included tonsillar lymphoid tissue for TOP2A and MCM2 and spleen for BUB1B protein, per manufacturer's recommendations. For negative controls, the respective primary antibodies were replaced by commercially available nonimmunized normal serum. Both types of controls showed satisfactory results. 

### 2.4. Scoring of Immunohistochemical Expression of TOP2A and MCM2 Proteins

**Figure 2:**
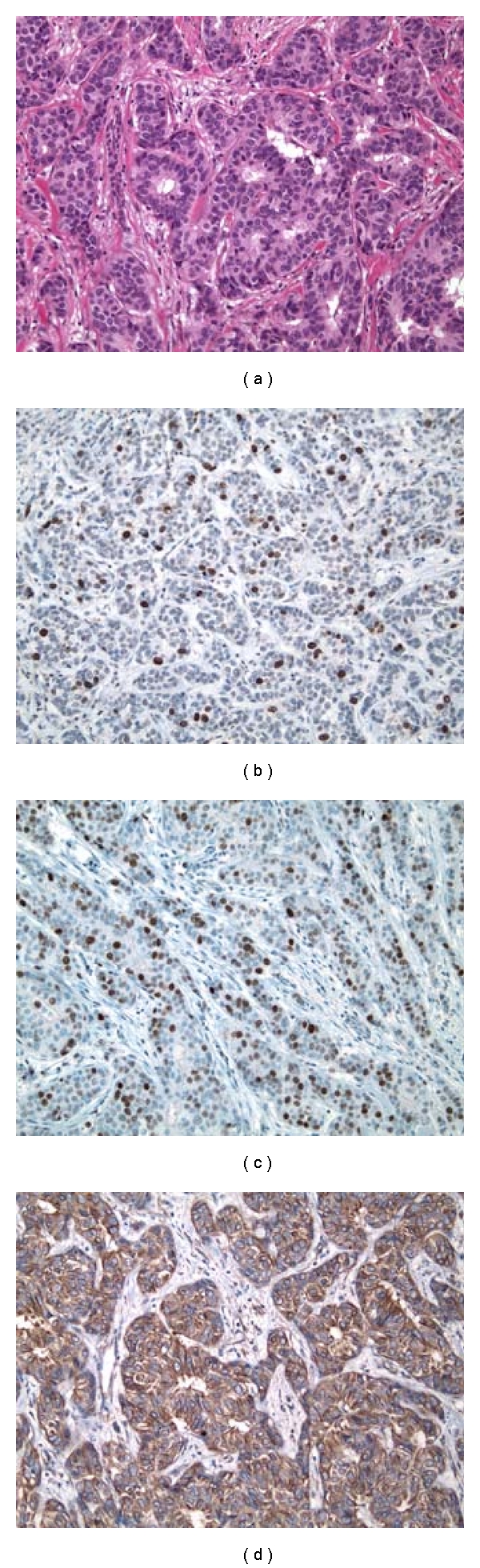
Serial archival sections representative of an IDC stained for H&E, TOP2A, MCM2 and BUB1B proteins. (a) Primary invasive ductal carcinoma (IDC) of the breast, grade 2, featuring focal tubular differentiation. (b, c, d) Distinct nuclear immunoreactivity for TOP2A marking the presence of cycling cells in about 15% of the infiltrating tumor cells, and for MCM2 marking the “licensed” population in about 1/3rd of the infiltrating tumor cells and diffuse cytoplasmic immunoreactivity (2+) with focal cell membrane accentuation for BUB1B protein (Immunoperoxidase staining (IMPOX staining); original magnifications 200x).

**Figure 3:**
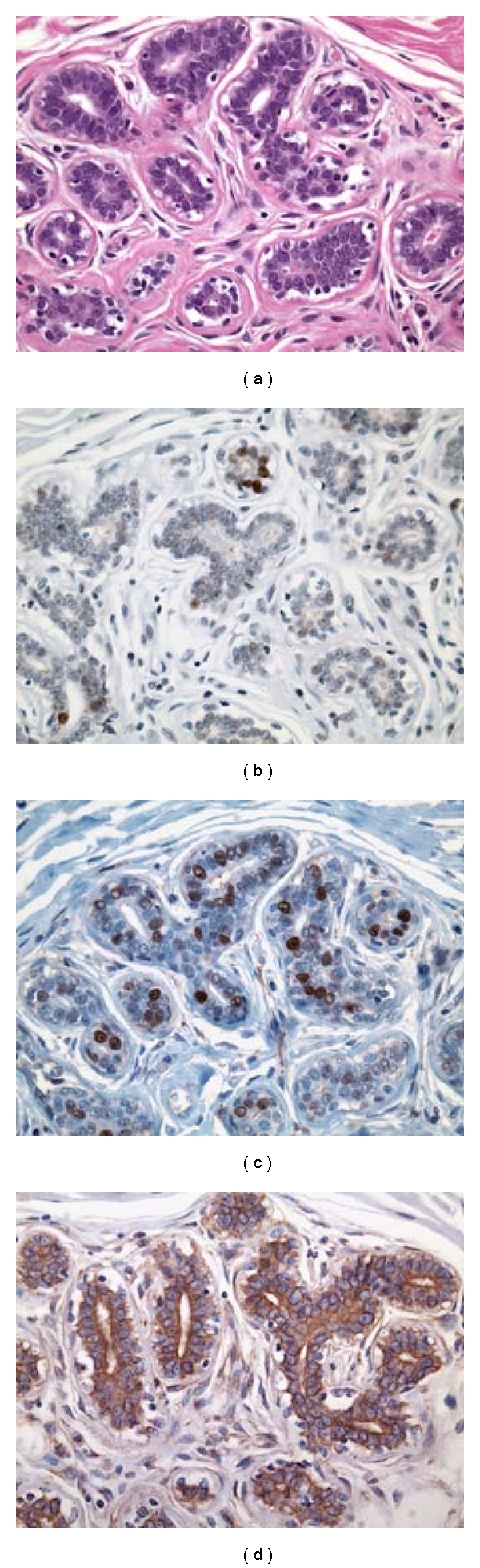
Serial archival sections representative of histologically normal breast tissues with high-grade molecular abnormality stained for H&E, TOP2A, MCM2 and BUB1B proteins. (a) Portion of a TDLU from a histologically normal breast tissue with high-grade molecular abnormality (Case  22, specimen 1495). Serial sections showing the same TDLU as in (a) with distinct nuclear immunoreactivity for TOP2A (b) and MCM2 (c) in the epithelial cell nuclei, and diffuse cytoplasmic immunoreactivity (2+) for BUB1B protein (d) in the mammary epithelial cells. (IMPOX staining; original magnifications 400x).

**Figure 4:**
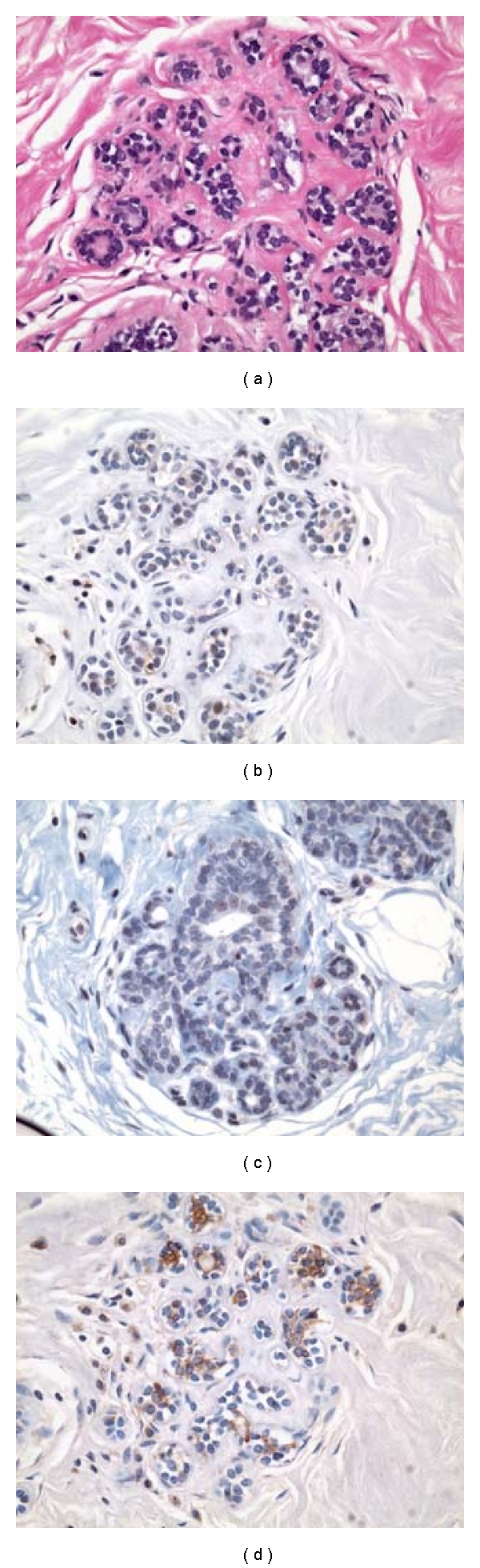
Serial archival sections representative of a histologically normal breast tissues with low-grade molecular abnormality stained for H&E, TOP2A, MCM2 and BUB1B proteins. (a) Portion of a TDLU from a molecularly low-risk, histologically normal breast tissue (Case  8, specimen 1481). Serial sections showing the same TDLU as in (a) without any expression of TOP2A (b) and MCM2 (c) in the epithelial cell nuclei. There is a focal cytoplasmic immunoreactivity (1+ to 2+) for BUB1B protein (d) in some of the mammary epithelial cells in this field. (IMPOX staining; original magnifications 200x).

The stained slides were evaluated by the breast pathologist on the study with extensive experience in immunohistochemistry (AN). Immunohistochemical staining for TOP2A and MCM2 was localized to the nuclei of the tumor cells and the normal breast epithelium, while the expression of BUB1B protein was localized to the cytoplasm of the tumor and normal breast epithelial cells. In order to calculate TOP2A and MCM2 nuclear staining indices in IDC tissue sections, up to 2000 tumor cells and in the case of histologically normal breast tissues (HNB-HGMA and HNB-LGMA) tissue sections up to 500 nonneoplastic breast epithelial cells were evaluated by absolute counting of positive (stained) and negative (unstained) cells in each section. TOP2A and MCM2 indices were recorded as per cent positive nuclei as previously described [13]. As outlined in the scheme published by Gonzalez et al. [14], these evaluations were made in the highest expression areas of the tumor and histologically normal breast tissues (Figures 2(b), 2(c), 3(b), 3(c), 4(b), and 4(c)). 

In the IDCs, both TOP2A- and MCM2-positive tumor cells were often more frequent at the peripheral/advancing edge of the tumor mass (Figures 2(b) and 2(c)), while in HNB tissues such cells were more randomly distributed within the epithelial lining of the mammary acini and ducts (Figures 3(b), 3(c), 4(b), and 4(c)). Overall, expression of these markers was observed predominantly in the mammary epithelial cells. In some areas, nuclear staining was also noted in an occasional myoepithelial cell in the outer layers of the benign mammary acini and ducts. Since myoepithelial expression was not a consistent finding in most benign mammary lobules, it was not included in the determination of TOP2A and MCM2 index. 

### 2.5. Scoring Immunohistochemical Expression of BUB1B Protein

**Figure 5:**
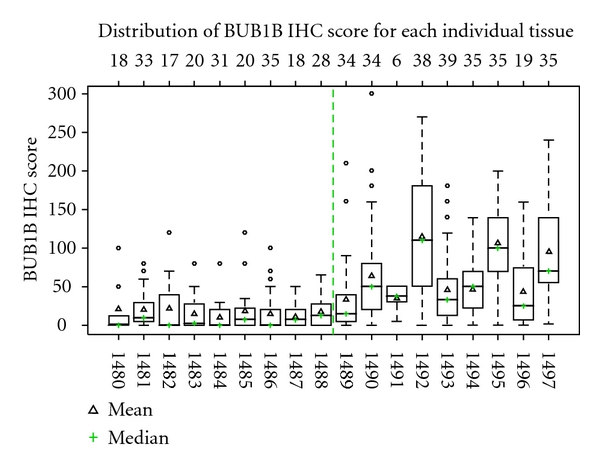
Whisker plot showing BUB1B protein expression scores for each individual histologically normal breast tissue with high-grade (*N* = 9) and low-grade molecular abnormality (*N* = 9) analyzed. The median BUB1B IHC score for each specimen is represented by horizontal lines and symbol +, while mean BUB1B IHC score is represented by Δ. Both mean and median expression scores for the HNB tissues with high-grade molecular abnormality on microarray (cases 1489–1497) are higher than those for the molecularly low-risk HNB tissues with low-grade abnormality on microarray (cases 1480–1488). Overall, there is a greater variation in the expression scores for the HNB tissues with high-grade molecular abnormality as compared to those with low-grade molecular abnormality (SD = 48.4 versus 24.8; *P* = .003). The top row reflects the number of TDLUs that were evaluated for IHC expression of BUB1B protein in the respective stained section, representing each histologically normal breast tissue specimen.

Since the intensity of cytoplasmic staining and the percentage of epithelial cells stained for BUB1B protein was variable from case to case and from lobule to lobule within the same case, a comprehensive immunohistochemical scoring method (*H*-score method) [15] was used for semiquantitative evaluation of BUB1B protein expression in the entire tumor and normal breast tissue sections: BUB1B protein staining intensity in the malignant (IDC) or benign breast epithelial cells was scored 0 when there was no cytoplasmic staining, 1+ for weak, 2+ for intermediate, and 3+ for strong cytoplasmic staining. The products of stained epithelial cells (%) and the respective staining intensity (0, 1+, 2+, 3+) were added to calculate the total BUB1B protein immunohistochemical staining score (*H*-score) for each IDC tissue and for each histologically normal TDLU in the HNB tissue section evaluated (Figure 5). The total number of TDLUs evaluated for immunohistochemical expression of TOP2A, MCM2, and BUB1B proteins in the HNB-HGMA and the HNB-LGMA tissue sections ranged from 6 (no other FFPE section with greater # of TDLUs was found on review of all archival slides on that case) up to a maximum of 39 TDLUs/section (Figure 5). The average number of TDLUs evaluated per HNB tissue section was 31 (range 6 to 39 TDLUs per section) per HNB-HGMA tissue section analyzed and 24 (range of 17–35 TDLUs per section) per HNB-LGMA tissue section (Table 3). For most precise interpretation of immunoreactive nuclei, the sections were assessed using the 20x objective. 

### 2.6. Differential Expression of TOP2A Protein in Independent Sets of Benign, Premalignant, and Cancerous Breast Tissues

Apart from cross-platform validation of 3 of our leading malignancy genes in archival HNB tissue samples, we further demonstrated the differential expression of TOP2A protein on independent test sets of Histologically normal breast tissues, including reduction mammoplasty samples, benign breast tissue from patients with and without synchronous breast cancer, and a set of DCIS and invasive breast carcinomas in a custom-designed breast TMA (Figure 10). 

### 2.7. Statistical Analysis

Analysis of variance was used to test the differences among the three sample groups (IDC, HNB-HGMA, and HNB-LGMA tissues) with the Tukey method to adjust for *P* value for pairwise comparison. This approach was used for analyzing the immunohistochemical expression data both from the FFPE sections and the breast TMA. Spearman correlation analysis was used to test the correlation between immunohistochemical expression of TOP2A, MCM2, and BUB1B proteins in the 3 sample groups. 

## 3. Results

### 3.1. Patient Characteristics

The 18 histologically normal breast tissues with low-grade (*N* = 9) and high-grade (*N* = 9) molecular abnormalities were identified based on the differential expression of our breast malignancy genes from a total of 143 frozen normal breast tissue samples collected from mastectomies in patients with invasive breast carcinoma, DCIS, or prophylactic mastectomies (prior microarray experiment). We then summarized pertinent clinicopathologic characteristics of these patients with HNB tissues with low-grade molecular abnormalities (Cases  7–15) and those with high-grade molecular abnormality (Cases  16–24) (Table 1). Four of the nine patients whose HNB tissues showed low-grade molecular abnormality on microarray had the final pathologic diagnosis of IDC, 4 had only DCIS, and 1 had mucinous carcinoma. Of nine patients whose HNB tissues showed high-grade molecular abnormality on microarray, two patients had the final pathologic diagnosis of IDC, one tubular carcinoma, one adenoid cystic carcinoma, one invasive lobular carcinoma, one papillary intracystic carcinoma, 2 DCIS, and one patient had no histologic evidence of malignancy in the prophylactic mastectomy specimen, despite thorough sampling. The last patient underwent prophylactic bilateral mastectomy because of strong family history of breast cancer and had tested positive for the BRCA1 gene. 

Mean age for the patient groups with IDCs, HNB-HGMA, and HNB-LGMA tissues was 63, 50, and 55 years, respectively (Table 2). Based on the analysis of variance (ANOVA), the difference in the distribution of patient ages at the time of diagnosis of their breast cancers (and collection of histologically normal tissues for the current analysis) was not statistically significant (*P* = .29). Since most patients whose normal breast tissues were found to exhibit HGMA or LGMA on prior microarray analysis [11] were peri-menopausal, the differential expression of TOP2A, MCM2, and BUB1B proteins (proliferation gene products) in this validation study is unlikely to be due to proliferative effect of estrogen on the normal/benign breast tissues analyzed. 

### 3.2. TOP2A, MCM2, and BUB1B Protein Immunohistochemistry

#### 3.2.1. Localization of Immunohistochemical Staining

TOP2A and MCM2 immunostaining was localized to the nuclei of the tumor cells (Figures 2(b) and 2(c)) and benign mammary epithelium (Figures 3(b) and 3(c)), while BUB1B protein immunostaining was cytoplasmic (Figures 2(d) and 3(d)), as has been demonstrated in a variety of normal human tissues [16]. In addition to cytoplasmic localization, an accentuation of BUB1B immunostaining (Figure 2(d)) was notable in cell membranes in some of the cases. Overall, a large proportion of tumor cells in the IDCs demonstrated a distinct nuclear staining for TOP2A (Figure 2(b) and MCM2 proteins (Figure 2(c)) and cytoplasmic staining for BUB1B protein (Figure 2(d)). However, the expression of these 3 biomarker proteins was found in smaller proportions of the epithelial cells lining the TDLUs present in the HNB-HGMA (Figures 3(b), 3(c), and 3(d)) and HNB-LGMA (Figures 4(b), 4(c), 4(d)) tissues analyzed. 

#### 3.2.2. TOP2A Protein Expression in IDCs and Histologically Normal Breast Tissues with High-Grade and Low-Grade Molecular Abnormality on Microarray

**Figure 6:**
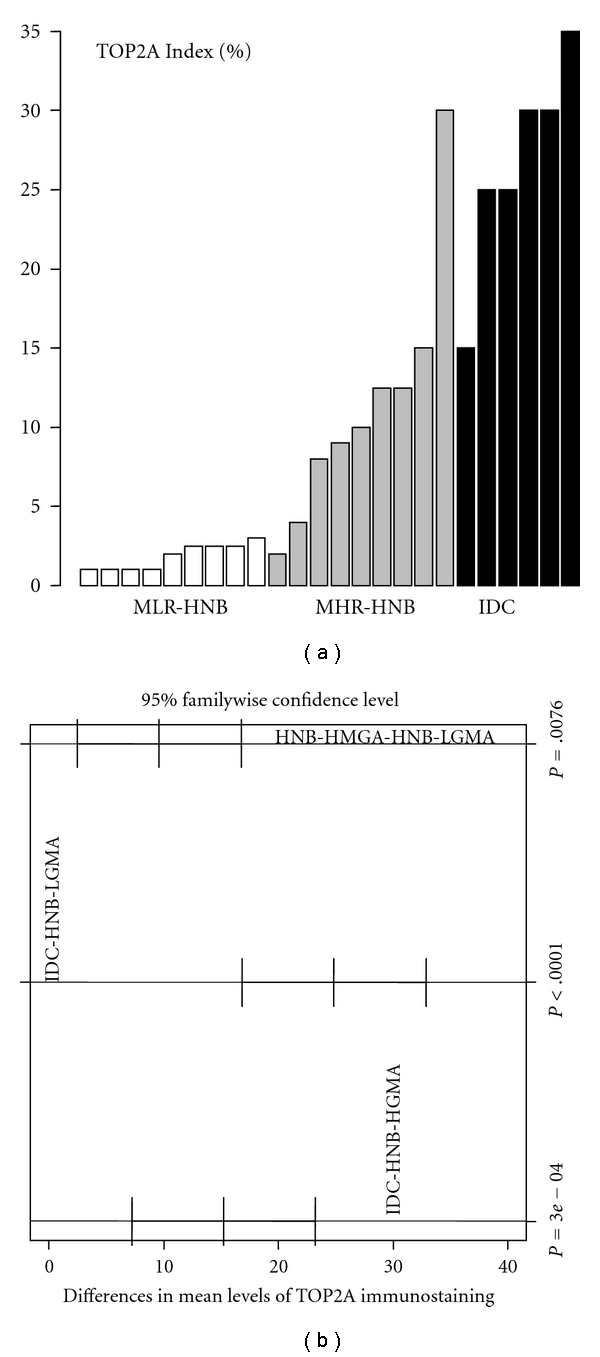
Immunohistochemical expresssion of TOP2A protein in HNB tissues with low-grade and high-grade molecular abnormalities and in IDCs. There is an obvious trend toward increasing expression from HNB tissues with low-grade molecular abnormality (white bars) to those with high-grade molecular abnormality (gray bars), and the IDCs (black bars), thus providing evidence for cross-platform validation of our original expression profiling data for TOP2A at the protein level. (a) Is the specimen-wise distribution of immunohistochemical expression of *TOP2A* for the HNB tissues with low-grade and high-grade molecular abnormality and IDC groups. (b) is the pairwise comparison of TOP2A immunostaining among the three groups. For each comparison (e.g., IDC versus normal), a mean difference with a 95% confidence interval (95% CI) is displayed to examine whether the difference is statistically significant (A 95% CI deviated away from 0 is statistically significant). The adjusted *P* value for each comparison, based on Tukey method, is shown.

Expression of TOP2A was nuclear both in the tumor cells (Figure 2(b)) and in the acinar and ductal epithelial cells present in the histologically normal breast tissues with high-grade (Figure 3(b)) and low-grade (Figure 4(b)) molecular abnormality. Mean TOP2A nuclear staining index values for IDCs and histologically normal breast tissues with high-grade and low-grade molecular abnormality were 27, 11, and 2, respectively. Compared to HNB tissues with low-grade molecular abnormality, TOP2A expression in HNB tissues with high-grade molecular abnormality was significantly higher, both in terms of absolute (Table 4) and mean (Table 3, Figure 6) TOP2A expression indices, thus validating our TOP2A gene expression data from frozen to archival histologically normal breast tissues at the protein level. 

**Figure 7:**
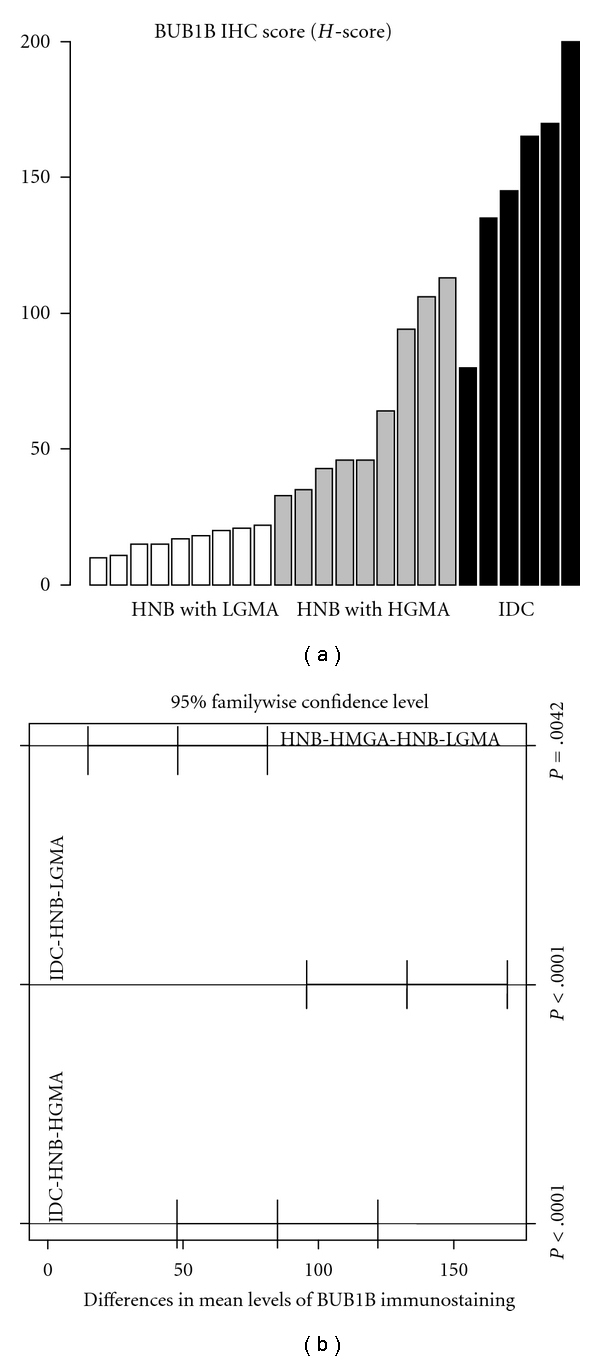
Immunohistochemical expression of BUB1B protein in HNB tissues with low-grade and high-grade molecular abnormalities and in IDCs. There is an obvious trend toward increasing expression from HNB tissues with low-grade molecular abnormality (white bars) to those with high-grade molecular abnormality (gray bars), and the IDCs (black bars), thus providing evidence for cross-platform validation of our original expression profiling data for BUB1B at the protein level. (a) Is the specimen-wise distribution of immuno-histochemical expression of *BUB1B* for the HNB tissues with low-grade and high-grade molecular abnormality and IDC groups. (b) Is the pairwise comparison of BUB1B immunostaining among the three groups. The adjusted *P* value for each comparison, based on Tukey method, is shown on (b).

MCM2 protein expression in IDCs and histologically normal breast tissues with high-grade and low-grade molecular abnormality on microarray Expression of MCM2 was nuclear both in the IDC cells (Figure 2(c)) and in the acinar and ductal epithelial cells present in the histologically normal breast tissues with high-grade (Figure 3(c)) and low-grade (Figure 4(c)) molecular abnormality. Mean MCM2 staining indexes for IDCs and histologically normal breast tissues with high-grade and low-grade molecular abnormality on microarray were 47, 20, and 4, respectively, showing higher immunohistochemical expression of MCM2 in the HNB tissues with high-grade molecular abnormalities compared to the HNB tissues with low-grade molecular abnormality (Table 4, Figure 7), thus validating the same trend as was evident in our gene expression data. While the majority of cases in HNB tissues with low-grade molecular abnormality had MCM2 index of 1-2%, 2 of the cases (Case #s 9 and 14) (Table 4) had higher MCM2 indices (12% and 8%, resp.), closer to the MCM2 index of some of the HNB tissues with high-grade molecular abnormality, suggesting that there may be a degree of heterogeneity in the expression of MCM2 protein in HNB tissues. 

#### 3.2.3. BUB1B Protein Expression in IDCs and Histologically Normal Breast Tissues with High-Grade and Low-Grade Molecular Abnormality on Microarray

Mean BUB1B protein cytoplasmic staining scores for IDCs and histologically normal breast tissues with high-grade and low-grade molecular abnormality on microarray were 149, 68, and 17, respectively (Table 3). As compared to low-risk normal breast tissues, this pattern of significantly higher immunohistochemical expression of BUB1B protein in histologically normal breast tissues with high-grade molecular abnormality as compared to low-grade molecular abnormality on microarray confirms the gene expression trends observed on microarray, thus validating our BUB1B RNA expression data at the protein level. Figure 5 shows the distribution of expression of BUB1B protein in the two molecular sets of histologically normal breast tissues. The histologically normal breast tissues with high-grade molecular abnormality had greater number of TDLUs available for evaluation per individual BUB1B protein-stained section than the HNB tissues with low-grade molecular abnormality on microarray molecularly low-risk group (the average number of breast lobules evaluated was 31 versus 24, resp.), but this difference was not statistically different (*P* = .18). 

#### 3.2.4. Differential Expression of TOP2A, MCM2, and BUB1B Proteins in IDCs and Molecularly High-Risk and Low-Risk, Histologically Normal Breast Tissues

**Figure 8:**
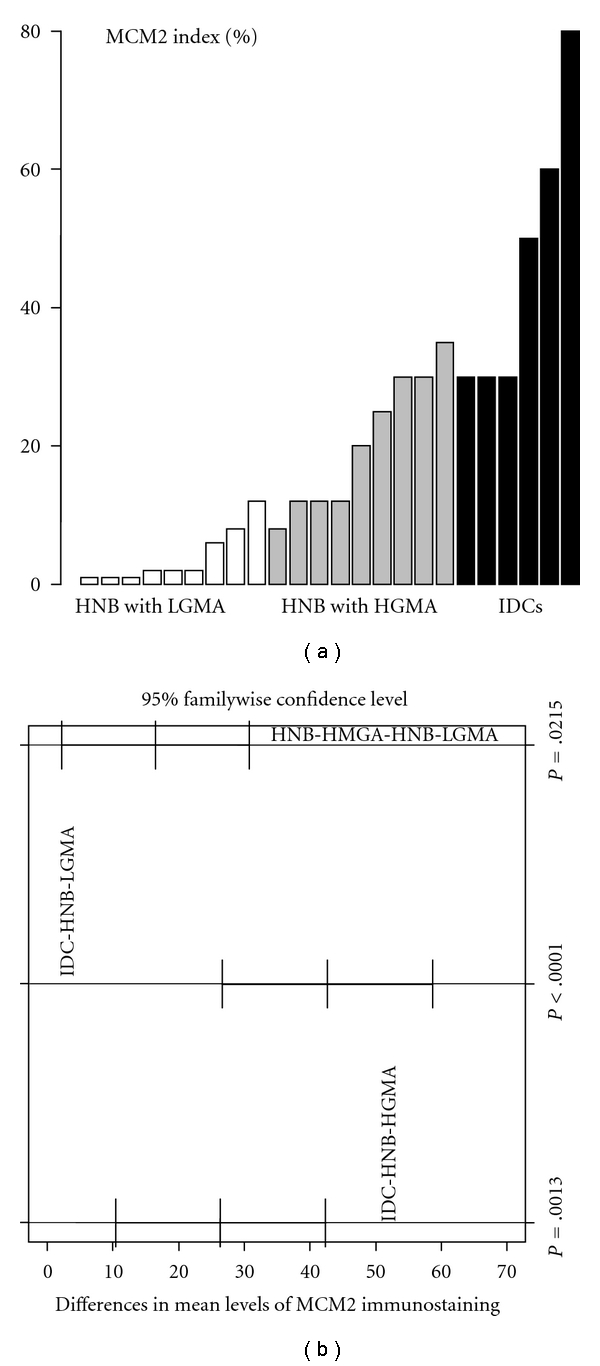
Immunohistochemical expresssion of MCM2 protein in HNB tissues with low-grade and high-grade molecular abnormalities and in IDCs. There is an obvious trend toward increasing expression from HNB tissues with low-grade molecular abnormality (white bars) to those with high-grade molecular abnormality (gray bars), and the IDCs (black bars), thus providing evidence for cross-platform validation of our original expression profiling data for MCM2 at the protein level. (a) Is the specimen-wise distribution of immunohistochemical expression of MCM2 for the HNB tissues with low-grade and high-grade molecular abnormality and IDC groups. (b) Is the pairwise comparison of MCM2 immunostaining among the three groups. The adjusted *P* value for each comparison, based on Tukey method, is shown on (b).

The immunohistochemical expression scores for TOP2A, MCM2, and BUB1B protein in the HNB-HGMA tissues were in the intermediate range between the higher scores (expression) for the IDCs and the lower scores (expression) for the HNB-LGMA tissues (Tables 3 and 4). In fact, for all 3 marker proteins, we observed a trend toward increasing immunohistochemical expression (TOP2A and MCM2 indices and BUB1B protein *H*-scores) from HNB-LGMA to HNB-HGMA tissues to the IDC tissues analyzed (Figures 6, 7, and 8). Analysis of variance showed that the differences in the immunohistochemical expression scores for TOP2A, MCM2, and BUB1B protein for the three types of tissues were statistically significant (*P* < .005 for TOP2A and BUB1B protein, and *P* < .05 for MCM2 for each pairwise comparison using the Tukey method). The differences in expression of these markers for individual pairs (and respective *P* values) are shown in Figures 6, 7, and 8. Furthermore, in comparing the HNB tissues with low-grade and high-grade molecular abnormality on microarray, the immunohistochemical expression of these 3 marker proteins was highly correlated (Spearman correlation ranges 0.84–0.90 with *P*  value < .0001: *r* = 0.84 for TOP2A versus BUB1B, *r* = 0.9 for TOP2A versus MCM2, and *r* = 0.88 for BUB1B versus MCM2). Taken together, these results validate our microarray expression data for TOP2A, MCM2, and BUB1B at the protein level in archival histologically normal breast tissues. 

#### 3.2.5. Pathologic Characteristics of the Cases on Breast TMA Stained for TOP2A

In order to further validate the differential expression of TOP2A protein in various benign, atypical, premalignant, and cancerous breast tissues, we immunostained a breast TMA for TOP2A, using the same IHC protocol as outlined above. The various groups of breast lesions represented on this TMA were as follows.

**Benign Lesions (*N* = 15)**

In this group seven adult females had undergone unilateral or bilateral reduction mammoplasty (RM). Others underwent diagnostic breast tissue sampling. Final pathologic evaluation showed histologically normal breast tissues with areas of benign breast disease (BBD) (*N* = 10), BBD with focal ductal hyperplasia (FDH) (*N* = 2), intraductal papilloma (*N* = 1), BBD with focus of atypical lobular hyperplasia (ALH) (*N* = 1), and BBD with focal fibroadenomatoid hyperplasia (*N* = 1).

**Atypical Ductal Hyperplasia (ADH) without Invasive Breast Carcinoma (*N* = 9)**

All specimens in this group showed BBD with foci of ADH. In addition, six (66%) cases showed columnar cell change and four (44%) had atypical lobular hyperplasia. There was one case with pseudoangiomatous stromal hyperplasia (PASH) and one case with an intraductal papilloma.

**ADH with Ipsilateral Invasive or In Situ Breast Carcinoma (*N* = 8)**

All of these cases showed ADH. In addition, three cases showed areas of invasive ductal carcinoma (IDC) while 5 cases had ductal carcinoma in situ (DCIS), 2 cases showed focal columnar cell change, and one of them also had an intraductal papilloma with atypia, a radial scar, and a fibroadenoma.

**Ductal Carcinoma In Situ (DCIS) (*N* = 15)**

Of the fifteen specimens in this group, 14 (93%) were intermediate to high-nuclear grade DCIS and one low-nuclear grade DCIS. Among these two specimens had areas of adenosis, focal ductal hyperplasia, PASH, and a fibroadenoma in addition.

**Invasive Ductal Breast Carcinomas (*N* = 20)**

These were histologically confirmed IDCs, of which 2 cases also had focal DCIS, intermediate to high nuclear grade. One IDC showed focal mucinous differentiation.

#### 3.2.6. Differential Expression of TOP2A Protein in Benign, Atypical, and Premalignant, and Cancerous Breast Tissues

**Figure 9:**
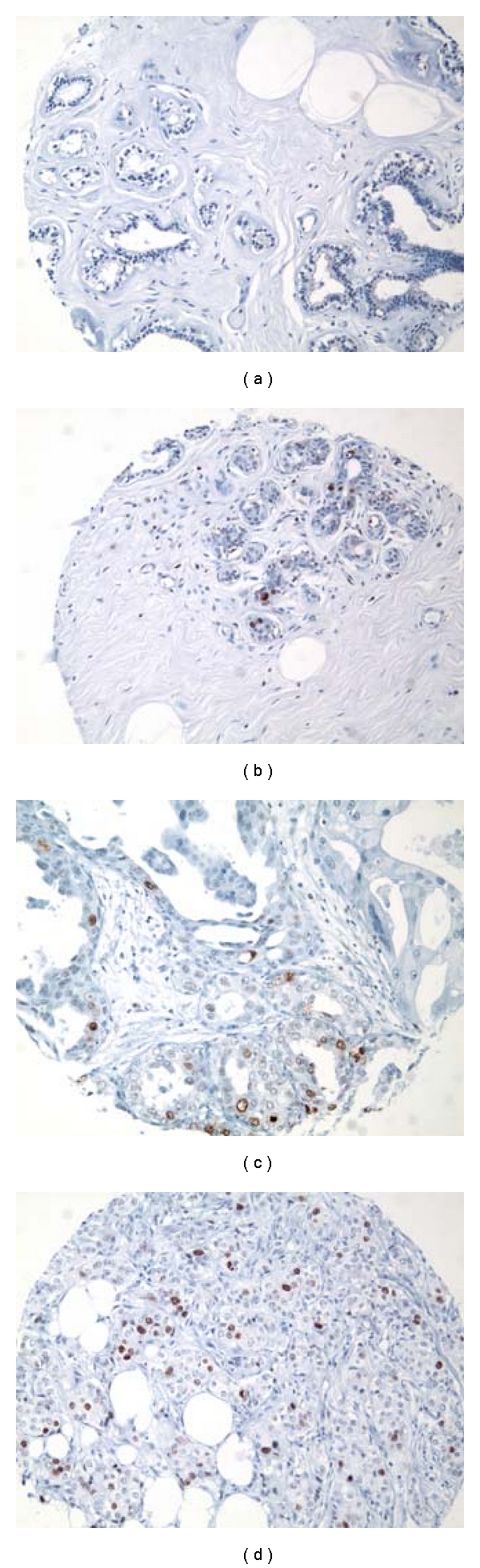
Immunohistochemical expression of TOP2A protein. (a) Histologically normal breast tissue from a reduction mammoplasty (RM) case featuring lack of nuclear expression of TOP2A in the epithelial cells lining a normal TDLU. (b) Histologically normal breast tissue from a patient with synchronous breast cancer showing positive nuclear staining in 4-5% of the mammary epithelial cells-higher TOP2A expression than the HNB tissues from a reduction mammoplasty case illustrated in (a). (c-d) A larger proportion of epithelial cells are immunoreactive for nuclear TOP2A protein in ductal carcinoma in situ (DCIS) and in the invasive ductal carcinoma (IDC) infiltrating the mammary fat. These cases illustrate an obvious increase in TOP2A protein expression from the lowest risk specimen from a reduction mammoplasty case (a), to the higher-risk specimens (c) and (d) (IMPOX staining for TOP2A; original magnifications 200x).

**Figure 10:**
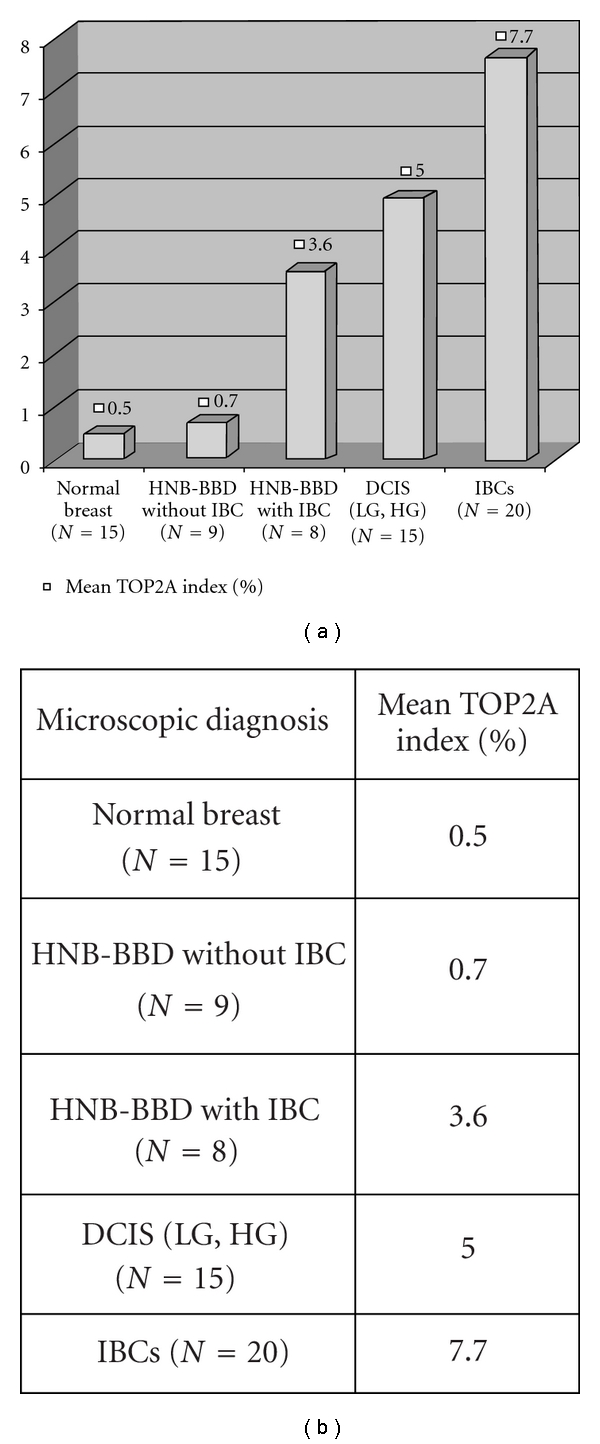
Mean TOP2A index in independent test sets of histologically normal breast (including reduction mammoplasty tissues), histologically normal and benign breast tissues from patients without and with synchronous cancer, DCIS and invasive breast carcinoma tissues. There is an obvious trend toward increasing TOP2A expression from benign to malignant breast tissues.

We found a striking trend toward increasing expression of TOP2A protein in this independent test set of histologically normal and benign breast tissues, ADH with or without synchronous invasive breast carcinoma, DCIS and invasive ductal breast carcinoma tissues, represented on the breast TMA. These results provide further validation of increasing expression of TOP2A protein along the histologic continuum of various breast lesions from benign to premalignant to invasive breast carcinomas it's (Figures 9(a), 9(b), 9(c), and 9(d)). For these specimen types, TOP2A protein expression data are summarized in Figure 10. 

## 4. Discussion

There is increasing evidence to support the hypothesis that histologically normal breast tissues contain genetic and epigenetic abnormalities that render them more susceptible to neoplastic transformation and that they might be detected through molecular analyses. In patients with sporadic breast cancer, abnormalities of breast cancer susceptibility genes, including TP53, BRCA1, and BRCA2, have been identified in tumor tissue, and also in histologically normal TDLUs adjacent to carcinoma [17]. In a recent study, Larson et al. found a threefold increase in allelic imbalance (AI) in histologically normal breast tissue from sporadic breast cancer patients and BRCA1 gene mutation carriers as compared to women who underwent reduction mammoplasty [5], suggesting that these genetic abnormalities may be contributing to the risk of development of malignancy. More recently, altered telomeres and unbalanced allelic loci (markers of genetic instability) were found both in human breast cancers and in surrounding histologically normal breast tissues [7]. These findings provide further support to the “cancer field effect” concept recognizing the presence of genetically aberrant cells that may represent high risk cell populations within the histologically normal breast tissues. In a more recent study, [10] elucidated the molecular differences between histologically normal breast tissue from breast cancer patients and reduction mammoplasty controls and found a number of global gene expression abnormalities in the HNB tissues [10]. 

Using specific epigenetic biomarkers, we have previously mapped a number of DNA methylation changes in histologically normal breast tissues as a potential explanation as to why histologically normal breast tissues are at risk for local recurrence after surgical therapy for breast cancer [6]. We recently developed a 117-gene signature by comparing the gene expression profiles of a large prospective cohort of frozen invasive ductal breast carcinoma (IDC) and histologically normal breast tissues (HNB) from breast cancer patients [11]. This signature was first cross-validated on HNB tissues using qPCR including external validation on previously published datasets [11]. We then used our 117-gene malignancy signature to classify eighteen histologically normal breast tissues with high-grade and low-grade molecular abnormality, based on the level of expression of our top malignancy genes. The leading candidate genes in our malignancy-risk signature were proliferation genes, including TOP2A, MCM2, and BUB1B. 

Here we present the results of cross-platform immunohistochemical validation of these candidate malignancy gene products (TOP2A, MCM2, and BUB1B proteins) on archival histological normal breast tissue sections from the mastectomies of the two patient groups in the original microarray experiment (those with HNB tissues with high-grade and low-grade molecular abnormalities). These candidate biomarkers were selected for validation based on the gene expression data and the availability of commercially available antibodies and to further investigate their usefulness as biomarkers of molecular abnormalities in histologically normal and benign breast tissues. We further confirmed the increasing expression of one of our malignancy-risk gene products in the present analysis on independent test sets of histologically normal breast tissues including reduction mammoplasty samples, which mostly represent the specimens with lowest risk of breast malignancy, histologically normal/benign breast tissues from patients with and without synchronous breast cancer and a set of DCIS and invasive breast carcinomas (IDCs) using a custom-designed breast TMA (Figure 10). 

One of our leading malignancy risk genes identified on microarray analysis of the histologically normal breast tissues was topoisomerase II alpha (TOP2A). TOP2A is a key enzyme in regulating various chromosomal events during tumor cell replication. It is one of the markers of cell proliferation in human breast cancer [18]. It is also the molecular target for topo II-inhibitors, including anthracyclines (doxorubicin, epirubicin, daunorubicin, idarubicin), epipodophyllotoxins (etoposide, teniposide), actinomycin, mitoxantrone, and others [19–21]. The TOP2A gene is located adjacent to the HER-2 oncogene at the chromosome location 17q12q21 and is either amplified or deleted in breast cancer, with or without HER-2 amplification. Recent evidence suggests that amplification or deletion of TOP2A gene may account for sensitivity or resistance to *topo II*-inhibitor (anthracycline) therapy in breast cancer [21]. However, little is known about the role of TOP2A in histologically normal/benign breast tissues. 

We identified TOP2A as a part of the malignancy-risk signature on our microarray experiment and, in this study, validated its expression at protein level as a potential biomarker of risk of malignancy in histologically normal breast tissues. In the archival sections from histologically normal breast tissues with high-grade and low-grade molecular abnormalities, we evaluated a large number of “morphologically normal TDLUs” and found that the level of expression of TOP2A protein in HNB tissues with high-grade molecular abnormality on microarray was intermediate between the expression levels in the HNB tissues with low-grade molecular abnormality on microarray and invasive ductal breast carcinoma tissues analyzed. Furthermore, the differences in the TOP2A expression levels between the two subsets of molecularly abnormal histologically normal breast tissues and IDCs were statistically significant. Since amplification of TOP2A gene leads to the overexpression of the TOP2A protein and better response to anthracycline therapy [22], while deletion of TOP2A gene leads to marked reduction in the expression of TOP2A protein and primary chemoresistance to TOP2 inhibitor drugs [23], our findings in histologically normal breast tissues, if clinically validated in larger series of histologically normal and benign breast tissues, may have potential implications for future chemopreventive trials in patients with various atypical and pre-malignant breast lesions. 

Since TOP2A amplified tumor cells tend to be sensitive to topo-II inhibitor therapy while TOP2A deleted tumor cells tend to be resistant to anthracycline chemotherapy [21], the overall response of a given breast cancer case will depend on the relative proportions of the 2 cell types. Furthermore, in locally advanced breast cancer, TOP2A levels in the primary tumor have been associated with greater tumor response to anthracycline therapy. It is, therefore, conceivable that in the case of molecularly abnormal histologically normal breast tissues increased expression of TOP2A may not only serve as a molecular biomarker of malignancy, but may also be potentially predictive of chemosensitivity to TOP2A inhibitors, in order to repress proliferation and subsequent transformation. These aspects merit further investigation on larger series of histologically normal and benign breast tissues.

MCM family of proteins are a novel class of proliferation markers, of which MCM2 is part of the prereplicative complex (pre-RC) that is assembled at the site of future DNA replication during the G1 phase to allow genome replication in the subsequent S phase. High-MCM2 index has been shown to correlate with high Ki-67 labeling [24] and has been shown to be a prognostic marker in a variety of human malignancies, including cancers of the esophagus, prostate, stomach and in diffuse large B-cell lymphoma [24–28]. In breast cancers, it appears to be a strong independent prognostic marker and the degree of MCM2 immunoreactivity has been correlated with high histologic grade [14, 29, 30]. In normal breast epithelium MCM2 has been shown to be a more sensitive marker of proliferation than the widely used proliferation marker, Ki-67 [14, 31], since it stains both the cycling cells and also the noncycling cells with proliferative potential [32]. However, not much is known regarding the association between MCM2 expression in normal and benign breast tissues. 

In our malignancy-gene signature, MCM2 was one of the leading malignancy-associated genes in a set of histologically normal breast tissues from peri-menopausal beast cancer patients. In this study, using the standard immunohistochemical approach, we have observed that the MCM2 index in HNB tissues with high-grade molecular abnormality was in the intermediate range between IDCs and HNB tissues with low-grade molecular abnormality, thus validating the overexpression of MCM2 protein in the set of HNB tissues that were showed high-grade molecular abnormality on our original microarray data analysis. 

In this study, we found expression of MCM2 protein in all of our cases of histologically normal breast tissues. Considering all of our normal breast samples together, the observed MCM2 index ranged from 1% to 35%. This wider variation is a reflection of an inherent case selection bias in our study, since we selected the 2 subsets of histologically normal breast tissues (with high- and low-grade molecular abnormality) based on differential expression of our malignancy- (proliferation-) associated genes. In a set of normal breast tissues from reduction mammoplasties, Shetty et al. found a median MCM2 expression of 35% [31]. This high level of expression is comparable to the highest levels of MCM2 expression in the HNB tissues with high-grade molecular abnormality in our study. Although normal breast tissues in the above study [31] were from the lowest risk specimens (reduction mammoplasties), a probable explanation for higher MCM2 indices in their study was premenopausal status of their patients, since estrogens are known to be a major promoter of proliferation in normal breast epithelium [33]. On the contrary, in our study it is unlikely that the higher MCM2 and other proliferation biomarkers (TOP2A and BUB1B) in the molecularly abnormal breast tissue samples were due to hormonal (estrogen) milieu of the patients studied, since both sets of HNB tissues (with high- and low-grade molecular abnormalities) in our study were from perimenopausal patients without any significant statistical difference in their ages. Therefore, a higher MCM2 expression in histologically normal breast tissues in our study is most likely a true molecular biomarker of malignancy rather than an estrogen-driven phenomenon. 

In another recent study of benign breast tissues from 30 patients who underwent lumpectomy for fibrocystic changes, ductal hyperplasia, and fibroadenomas, the overall MCM2 labeling index was from 0% to 12% [30]. This pattern of expression is comparable to the HNB tissues with low-grade molecularl abnormality in our study. In our preliminary analysis, we did not find an obvious and linear relationship between the expression of MCM2 and the histologically defined risk categories of benign breast disease. Interestingly, we found higher expression of MCM2 and other proliferation marker proteins in histologically normal TDLUs as compared to the adjacent hyperplastic lobular units and incidental areas of epithelial hyperplasia on the same histologic sections of HNB tissues. This suggests that the expression of our malignancy-associated proliferation marker proteins may be independent of the various histologic risk categories of benign breast disease as was originally defined on the basis of degree of epithelial proliferation and cytologic atypia [34–36], and subsequently endorsed at a Consensus Conference of the College of American Pathologists [37]. We are intrigued by this finding and would like to extend this into a systematic analysis of the expression of these biomarkers and various benign and preneoplastic histologic correlates of breast cancer risk, as they have been recognized in the literature over the years [35, 38–42].

BUB1B protein is a mitotic checkpoint kinase required for cell mitotic divisions following severe cell damage or mutation [43, 44]. It has been associated with cell proliferation both in neoplastic and nonneoplastic tissues [16, 45, 46] and also with tumor progression [47, 48]. BUB1B is also a cellular target of synuclein-gamma (SNCG, also known as breast cancer specific gene 1), with which it may interact to inactivate the mitotic checkpoint, and contribute to resistance of beast cancer cells to microtubule inhibitors. Recently, a strong association has been found between BUB1B and other mitotic checkpoint genes and breast cancer risk [49]. Furthermore, checkpoint genes, including BUB1B, are expressed at high levels in breast cancer, both at transcriptional (RNA) and translational (protein) levels [50].

In this study, we have validated overexpression of BUB1B protein in histologically normal breast tissues that were found to be molecularly abnormal on microarray, thus validating our prior microarray and real-time PCR results. Our study suggests that BUB1B overexpression may be a new immunohistochemical biomarker of malignancy in histologically normal breast tissues. It will also be interesting to investigate the role of BUB1B overexpression as a potential therapeutic target for microtubule inhibitors and an immunohistochemical biomarker of predictive of chemosensitivity of atypical and pre-malignant breast lesions to these antimitotic agents. 

Expression of hormone receptors is an established predictor of response of breast cancer to hormonal therapy in breast cancer, but markers predictive of chemosensitivity of breast cancer are less well defined [51]. In addition, markers that could predict effective prevention of human breast cancer in high-risk patient populations are largely unknown. Among the proliferation-associated proteins (TOP2A, MCM2, and BUB1B) that we have studied immunohistochemically on a set of IDCs and validated as immunohistochemical biomarkers of malignancy in histologically normal breast tissues, TOP2A and BUB1B protein are also known targets of established chemotherapeutic approaches in breast cancer: anthracyclines and antimicrotubule therapies, respectively. It will, therefore, also be interesting to explore how these biomarkers can be utilized as predictors of breast cancer response to TOP2A and antimicrotubule inhibitors. 

## 5. Summary

To our knowledge, this is the first IHC-based analysis focusing on the pattern of coexpression of newer proliferation-associated proteins (TOP2A, MCM2, and BUB1B) in histologically normal breast tissues. In continuation of our prior transcriptional validation using qPCR, in this immunohistochemical validation study, we have demonstrated significantly higher expression of TOP2A, MCM2, and BUB1B proteins in a set of histologically normal breast tissues that were found to have high-grade molecular abnormality on microarray, based on our novel 117-gene malignancy signature. Taken together, these data further validate our leading candidate malignancy-risk genes (TOP2A, MCM2, and BUB1B) at the protein level. In addition, we have shown incremental expression of TOP2A protein on independent test sets of histologically normal breast tissues (including reduction mammoplasty samples), histologically normal/benign breast tissue from patients with and without synchronous breast cancer, and a set of DCIS and invasive breast carcinomas using a custom breast TMA. This study reveals new information about the coexpression of TOP2A, MCM2, and BUB1B proteins in histologically normal breast tissues and provide preliminary evidence to support further analyses of these proteins on larger series of histologically normal, benign, pre-malignant, and malignant breast tissues. Specifically, determination of TOP2A, MCM2, and BUB1B protein expression status may provide an objective tool to evaluate of the molecular signature of malignancy in histologically normal and benign breast tissues.

The immunohistochemical approach used here offered some distinct technical advantages over other techniques like qPCR or microarray: (1) combined assessment of the degree of expression (high versus low), microanatomical distribution (diffuse versus patchy), tissue (epithelial versus stromal), and subcellular (nuclear versus cytoplasmic) localization of the biomarker proteins in a given sample; (2) comparative evaluation of the relative expression of these marker proteins in histologically normal TDLUs and various incidental benign and pre-malignant breast lesions present in the same archival breast tissue sections. We do recognize one of the limitations of our study—the fewer numbers of histologically normal breast tissue analyzed. However, since we have successfully validated the expression of TOP2A, MCM2, and BUB1B proteins in HNB tissues with various grades of molecular abnormalities, we are in the process of now expanding our investigation to larger sample size and a wider range of benign pre-malignant and malignant breast tissues. 

## 6. Conclusions

The data presented in this technical validation study of a novel set of molecular biomarkers (TOP2A, MCM2, and BUB1B proteins) in histologically normal breast tissues confirms our microarray data at the protein level. We have also unraveled a preliminary association between the expression of these marker proteins and different stages of mammary carcinogenesis (histologically normal to benign to pre-malignant and fully invasive malignant breast tissues). Additional studies on larger selection of histologically normal, benign, and pre-malignant breast tissues are needed to fully explore the clinical utility of these biomarkers in the stratification of histologically normal breast and benign and premalignant breast lesions into those with various levels of molecular abnormalities. Such classification may potentially be predictive of response of various benign, atypical, and pre-malignant to targeted chemopreventive approaches. 

## Figures and Tables

**Table 1:** Clinicopathologic summary of IDC, molecularly high-risk and molecularly low-risk, histologically normal breast tissue specimens.

			Outside institution					Moffitt Cancer Center (MCC)							
Case no.	Tiisue core ID (frozen specimens for microarray)	Patient age (Years)	Side of breast sampled	Date of first-tissue diagnosis of BRCA	Surgical procedure at first-tissue diagnosis of BRCA; INA = Information not available	Pathologic diagnosis of BRCA	Date of surgical procedure at MCC	Side of breast sampled	Surgical procedure	Axillary LN dissection = AX; Sentinel LN dissection, axillary = SLN-AX; Not done = ND	Final pathologic diagnosis	Histologic grade (Invasive carcinoma)	Tumor size (cm)	DCIS	Other histopathologic findings (resected specimen)
1	X	41	Left	2001.04.10	Core biopsy	IDC, DCIS, high grade, comedo type	2005.08.04	Left	Lumpectomy	Intramammary lymph nodes	IDC	3	5	Present	X
1	X	41	Right	X	X	X	2005.08.04	Right	Lumpectomy	ND	IDC	3	4	Present	Skeletal muscle invasion, multifocal
2	X	38	Left	2005.07.01	Core biopsy	IDC	2005.08.03	Left	Modified radical mastectomy	SLN-AX	Papillary CA with a focus of IDC	3	7.5	Present	IDC focus 0.8 cm
3	X	68	Right	2005.07.20	INA	IDC	2005.08.09	Right	Total mastectomy	SLN-AX	IDC	3	2.2	Present	X
4	X	47	Left	2005.11.28	INA	IDC	2006.01.09	Left	Mastectomy	SLN-AX	IDC	3	2	Present	Focal ductal hyperplasia, radial scar, sclerosing adenosis, microcalcifications
4	X	47	Right	X	X	X	2006.01.09	Right	Mastectomy	SLN-AX	Fibroadenomatoid hyperplasia, florid ductal hyperplasia, sclerosing adenosis, microcalcifications	NA	NA	Absent	X
5	X	53	Right	2006.05.09	Core biopsy	IDC	2006.07.31	Right	Mastectomy	AX	Residual IDC	3	2.8	NS	involve dermis of skin/nipple
6	X	71	Left	2006.04.12	Core biopsy	Ductal & Lobular CA	2006.05.23	Left	Modified radical mastectomy	SLN-AX	IDC w/lobular features	3	4	Present	Tumor involves dermal angiolymphatic space in areola
7	1480	61	Right	2006.05.09	INA	IDC	2006.08.01	Right	Mastectomy	SLN-AX	Benign breast	NA	NA	Absent	ALH, proliferative fibrocystic changes
7	X	61	Left	X	X	X	2006.08.01	Left	Mastectomy	SLN-AX	IDC	2	2.6	Absent	
8	1481	86	Right	2006.07.31	Core biopsy	IDC	2006.08.29	Right	Total mastectomy	SLN-AX	IDC	3	8.5	Present	involve dermis of skin/nipple
9	1482	52	Left	2006.02.22	Core biopsy	IDC	2006.03.30	Left	Total mastectomy, skin-sparing	SLN-AX	IDC	2	1.5	Present	X
10	1483	80	Left	2005.06.13	Excisional biopsy	Mucinous Carcinoma	2005.08.16	Left	Total mastectomy	Not done	No residual carcinoma	NA	NA	Absent	X
11	1484	42	Left	2005.07.06	Core biopsy	DCIS solid type	2005.08.15	Left	Mastectomy	SLN-AX	Residual DCIS	NA	0.4	Present	DCIS, multifocal
11	X	42	Right	X	X	X	2005.08.15	Right	Mastectomy	SLN-AX	Benign breast	NA	NA	Absent	X
12	1485	52	Left	2006.03.31	Core biopsy	IDC	2006.05.30	Left	Total mastectomy	ND	No residual carcinoma	NA	NA	Absent	X
12	X	52	Right	X	X	X	2006.05.30	Right	Rt modified radical prophylactic mastectomy	AX	Benign breast	NA	NA	Absent	X
13	1486	67	Right	2006.05.02	Core biopsy	DCIS	2006.07.12	Right	Mastectomy	SLN-AX	DCIS	NA	1.2	Present	X
13	X	67	Left	X	X	X	2006.07.12	Left	prophylactic mastectomy	SLN-AX	Benign breast	NA	NA	Absent	X
14	1487	67	Left	2006.06.12	Core biopsy	DCIS	2006.07.31	Left	Mastectomy	SLN-AX	No residual DCIS	NA	NA	Absent	
14	X	67	Right	X	X	X	2006.07.31	Right	prophylactic mastectomy	SLN-AX	Benign breast	NA	NA	Absent	microcalcifications
15	1488	49	Left	2005.05.23	Excisional biopsy	DCIS	2005.08.02	Left	Total Mastectomy	SLN-AX	Residual DCIS	NA	X	Present	X
16	1489	49	Left	2005.05.27	Excisional biopsy, subareolar	Adenoid cystic CA	2005.06.14	Left	Total Mastectomy	SLN-AX	No residual carcinoma	NA	NA	Absent	ADH
16	X	49	Right	X	X	X	2005.06.14	Right	prophylactic mastectomy	SLN-AX	Benign breast	NA	NA	Absent	X
17	1490	39	Right	2005.04.26	Excisional biopsy	DCIS solid type	2005.06.29	Right	Mastectomy	SLN-AX	DCIS, multifocal	NA	2.2	Present	X
18	1491	85	Left	2005.06.30	Excisional biopsy	Intracystic CA	2005.07.28	Left	Mastectomy	SLN-AX	No residual carcinoma	NA	NA	Absent	ADH
19	1492	47	Left	2005.11.11	Needle-loc excisional biopsy	IDC	2006.05.02	Left	Skin-sparing total mastectomy	SLN-AX	IDC focal micropapillary features	3	2.5	Present	X
19	X	47	Right	2006.02.27	Needle core biopsy	Fibrocystic changes	2006.05.02	Right	Needle-loc excisional biopsy	SLN-AX	Adenoma w/adenomyoepitheliomatous features and focal atypia	NA	NA	Absent	Sclerosing adenosis, cystic/apocrine changes
20	1493	55	Right	2006.01.03	Cytology: Adenocarcinoma	IDC	2006.02.20	Right	Mastectomy	SLN-AX	IDC	3	2	NS	X
20	X	55	Left	X	X	X	2006.02.20	Left	Mastectomy	SLN-AX	ALH; No invasive carcinoma	NA	X	X	ALH involving lactiferous duct
21	1494	50	Right	2006.03.15	Core biopsy	IDC	2006.05.22	Right	Bilateral nipple sparing mastectomy	SLN-AX	No residual carcinoma	NA	NA	Absent	X
21	X	50	Left	X	X	X	2006.05.22	Left	As above	SLN-AX	Benign breast	NA	NA	Absent	X
22	1495	18	Right	NA	NA	No clinical evidence of invasive carcinoma	2006.06.19	Right	prophylactic mastectomy*	SLN-AX	Benign breast	NA	NA	Absent	X
22	X	18	Left	X	X	X	2006.06.19	Left	prophylactic mastectomy*	SLN-AX	Benign breast	NA	NA	Absent	X
23	1496	56	Right	2006.04.04	Unknown	DCIS	2006.06.15	Right	Bilateral mastectomy	SLN-AX	No residual DCIS	NA	NA	Absent	Ductal hyperplasia, microcalcifications
23	X	56	Left	X	X	X	2006.06.15	Left	Bilateral mastectomy	SLN-AX	Benign breast	NA	NA	Absent	Fat necrosis, microcalcifications
24	1497	48	Right	2006.06.27	Lumpectomy	IDC, ILC	2006.07.28	Right	Modified radical mastectomy	SLN-AX	No residual invasive carcinoma; LCIS involves nipple duct	NA	NA	Absent	X
24	X	48	Left	X	X	X	2006.07.28	Left	prophylactic mastectomy	ND	Benign breast	NA	NA	Absent	Focal secretory change

*Strong family history of breast cancer and patient tested positive for BRCA1 gene.

**Table 2:** Patient age distribution for IDC, molecularly high-risk and low-risk, histologically normal breast tissue groups.

	IDC patients	Patients with histologically normal breast tissues with low-grade molecular abnormality on microarray confirmed by IHC	Patients with histologically normal breast tissues with high-grade molecular abnormality on microarray confirmed by IHC
Mean age	63	55	50
Standard deviation	14.3	15.16	17.48
Total no. of cases	6	9	9

**Table 3:** Mean TOP2A and MCM2 indices and BUB1B protein expression scores in IDCs and molecularly high-risk and low-risk, histologically normal breast tissues.

Archival specimen type	Average no. of TDLUs evaluated/specimen (Range)	Mean TOP2A index (%) by IHC	Mean MCM2 index (%) by IHC	Mean BUB1B protein expression score (*H*-score) by IHC (Range)
IDCs (*N* = 6)	Not applicable	27 (15–35)	47 (30–80)	149 (80–200)
Histologically normal breast tissues with high-grade molecular abnormality (*N* = 9) on microarray	31 (6–39)	11 (2–30)	20 (8–35)	68 (33–113)
Histologically normal breast tissues with low-grade molecular abnormality (*N* = 9) on microarray	24 (17–35)	2 (1–3)	4 (1–12)	17 (10–22)
*P* value	.18	<.005	<.05	<.005

**Table 4:** TOP2A, MCM2, and BUB1B protein expression scores in IDCs, molecularly low-risk and molecularly high risk, histologically normal breast tissues.

Case no.	Breast tissue specimen category (based on gene expression profiling)	Histologic tumor type on initial biopsy/lumpectomy	Final pathologic diagnosis on mastectomy	Histopathologic findings on archival tissue sections selected for IHC validation	Topoisomerase II-alpha (TOP2A) index (%)	MCM2-index (%)	BUB1B protein expression score (*H*-score)
1	Carcinoma	IDC	IDC, DCIS	Invasive Cancer	25	30	170
2	Carcinoma	IDC	Invasive papillary CA with a focus of IDC	Invasive Cancer	30	30	135
3	Carcinoma	IDC	IDC	Invasive cancer	15	80	145
4	Carcinoma	IDC	IDC	Invasive Cancer	35	60	80
5	Carcinoma	IDC	IDC	Invasive Cancer	25	50	165
6	Carcinoma	IDC, ILC	IDC w/ lobular features	Invasive cancer	30	30	200

HNB tissues with low-grade molecular abnormality (HNB-LGMA)							

7	HNB-LGMA 1	IDC	IDC	Benign breast tissue	2.5	2	21
8	HNB-LGMA 2	IDC	IDC	Benign breast tissue	2.5	6	20
9	HNB-LGMA 3	IDC	IDC	Benign breast tissue	2.5	12	22
10	HNB-LGMA 4	Mucinous carcinoma	No residual mucinous carcinoma	Benign breast tissue	1	2	15
11	HNB-LGMA 5	DCIS	Residual DCIS, multifocal	Benign breast tissue	1	1	10
12	HNB-LGMA 6	IDC	No residual IDC	Benign breast tissue	1	1	18
13	HNB-LGMA 7	DCIS	Residual DCIS	Benign breast tissue	2	2	15
14	HNB-LGMA 8	DCIS	No residual DCIS	Benign breast tissue	3	8	11
15	HNB-LGMA 9	DCIS	Residual DCIS	Benign breast tissue	1	1	17

HNB tissues with high-grade molecular abnormality (HNB-HGMA)							

16	HNB-HGMA 1	Adenoid cystic carcinoma	No residual adenoid cystic carcinoma	Benign breast tissue	8	12	33
17	HNB-HGMA 2	DCIS	DCIS, multifocal	Benign breast tissue	12.5	20	64
18	HNB-HGMA 3	Intracystic carcinoma	No residual intracystic carcinoma	Benign breast tissue	12.5	8	35
19	HNB-HGMA 4	IDC	IDC focal papillary features	Benign breast tissue	9	30	113
20	HNB-HGMA 5	IDC	IDC	Benign breast tissue	2	12	46
21	HNB-HGMA 6	IDC	No residual IDC	Benign breast tissue	15	30	46
22	HNB-HGMA 7	No prior biosy performed	Benign breast tissue-patient BRCA1+, strong family history of BC	Benign breast tissue	10	25	106
23	HNB-HGMA 8	DCIS	No residual DCIS	Benign breast tissue	4	12	43
24	HNB-HGMA 9	IDC, ILC	No residual invasive carcinoma	Benign breast tissue	30	35	94

## Abbreviations

TOP2A: Topoisomerase 2 alpha
MCM2: Minichromosome maintenance protein 2
BUB1B: Benzimidazoles 1 homolog beta'
HNB: Histologically normal breast
TDLUs: Terminal duct lobular units
BB: Benign breast
HELUs: Hyperplastic enlarged lobular units
EH: Epithelial hyperplasia
FDH: Focal ductal hyperplasia
ADH: Atypical ductal hyperplasia
DCIS: Ductal carcinoma in situ
BC: Breast cancer
IDC: Invasive ductal carcinoma
TMA: Tissue microarray
qPCR: Real-time PCR
HNB-HGMA: Histologically normal breast with high-grade molecular abnormality
HNB-LGMA: Histologically normal breast with low-grade molecular abnormality
FFPE: Formalin fixed, paraffin-embedded
SLN-AX: Sentinel lymph node dissection, AXILLARY
Sd: Standard deviation.
